# Polyphenol oxidase depletion in *Nicotiana benthamiana* enhances recombinant protein purification and preserves native protein integrity

**DOI:** 10.1111/nph.71262

**Published:** 2026-05-24

**Authors:** Kaijie Zheng, Farnusch Kaschani, Emma C. Watts, Markus Kaiser, Renier A. L. van der Hoorn

**Affiliations:** ^1^ The Plant Chemetics Laboratory, Department of Biology University of Oxford OX1 3RB Oxford UK; ^2^ Chemical Biology, Faculty of Biology Universität Duisburg‐Essen, ZMB 45141 Essen Germany

**Keywords:** agroinfiltration, biotechnology, crosslinking, molecular pharming, *Nicotiana benthamiana*, polyphenol oxidase

## Abstract

- Agroinfiltration of *Nicotiana benthamiana* is widely used for recombinant protein production in plant science and molecular pharming, but enzymatic browning and native protein crosslinking during extraction may limit protein integrity and purification efficiency.
- We generated genome‐edited *N. benthamiana* lines lacking two polyphenol oxidases (PPOs) and analyzed protein integrity, enzymatic activity profiles, and recombinant protein purification under non‐denaturing extraction conditions.
- PPO‐deficient plants showed reduced browning and native protein crosslinking, preserved endogenous proteins at their predicted molecular weights, displayed increased detectable enzyme activities, and achieved a significantly higher recovery and improved purity of a transiently expressed recombinant protein.
- These findings identify PPO‐mediated oxidation as a major bottleneck during protein extraction and demonstrate that PPO depletion enhances recombinant protein purification while preserving native protein integrity.

## Graphical abstract

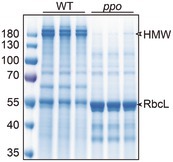

## 概括

- 在本氏烟草中进行农杆菌瞬时侵染是植物科学和分子医药农业中广泛使用的重组蛋白生产方法，但提取过程中发生的酶促褐变和内源蛋白交联可能会限制蛋白的完整性和纯化效率。
- 我们构建了敲除两个多酚氧化酶基因的本氏烟草株系，并在非变性提取条件下分析了蛋白质完整性、酶活性谱以及重组蛋白的纯化情况。
- *PPO*缺失突变体表现出褐化和内源蛋白交联程度的降低，内源蛋白在其预测分子量处得到保留，并且检测到更高的酶活性，此外在瞬时表达重组蛋白时实现了显著更高的回收率和更优的纯度。
- 这些结果表明，PPO介导的氧化是蛋白质提取过程中的一个主要瓶颈，同时表明去除PPO可以在保持内源蛋白完整性的同时，提高重组蛋白的纯化效果。

## Introduction

Agroinfiltration of *Nicotiana benthamiana* has become one of the most widely used transient expression platforms in plant science and molecular pharming, owing to its speed, scalability, low production cost, and ability to produce complex eukaryotic proteins with posttranslational modifications (Beritza *et al*., 2024a; Lawson *et al*., 2026; Bally *et al*., 2018; Golubova *et al*., 2024). This system is routinely used to study protein–protein interactions (Win *et al*., 2011), enzymatic activities (Dudley *et al*., 2022), protein structure (Lawson *et al*., 2026), and subcellular localization (Alamos *et al*., 2025) and has been successfully deployed for the production of vaccines (Ward *et al*., 2020, 2021), antibodies (Shanmugaraj *et al*., 2020; de Taeye *et al*., 2025), and other biopharmaceuticals (Busold *et al*., 2022, 2024; Kaldis *et al*., 2023; VanderBurgt *et al*., 2023), including plant‐based COVID‐19 vaccines (Charland *et al*., 2022; Hager *et al*., 2022).

Despite these advantages, the purification of recombinant proteins from agroinfiltrated *N. benthamiana* leaves remains a major bottleneck (Buyel, 2025). Leaf homogenization inevitably disrupts cellular compartmentalization, releasing phenolic compounds, chlorophyll (Chl), and a diverse array of endogenous enzymes that can compromise protein integrity, activity, and purity (Jutras *et al*., 2020; Buyel, 2025; Daduang *et al*., 2025; Lawson *et al*., 2026). One of the most prominent and widely observed problems during protein extraction from plant tissues is enzymatic browning, a process driven primarily by polyphenol oxidases (PPOs). PPOs are copper‐dependent oxidoreductases localized in chloroplasts that catalyze the oxidation of monophenols and *o*‐diphenols into highly reactive *o*‐quinones (Boeckx *et al*., 2015). These quinones can polymerize into brown, melanin‐like pigments and react nonspecifically with nucleophilic residues on proteins, leading to covalent crosslinking and loss of solubility (Sommer *et al*., 1994; Sui *et al*., 2023; Tang *et al*., 2023).

In fruits and vegetables, PPO‐mediated browning is well known to reduce food quality, and extensive efforts have been devoted to its suppression through breeding, genome editing, and chemical inhibition (Terefe *et al*., 2014; González *et al*., 2020; Ren *et al*., 2024; Zou *et al*., 2025). By contrast, the impact of PPO activity on recombinant protein extraction from plant expression systems has received comparatively little attention. Our recent work using virus‐induced gene silencing (VIGS) demonstrated that transient depletion of PPO transcripts in *N. benthamiana* suppresses enzymatic browning and native protein crosslinking in leaf extracts, resulting in greener extracts and improved protein recovery (Mahadevan *et al*., 2025). Notably, PPO depletion prevented the formation of high molecular weight (HMW) complexes of the large subunit of ribulose‐1,5‐bisphosphate carboxylase/oxygenase (RbcL), suggesting that PPO activity drives extensive native protein crosslinking during extraction (Mahadevan *et al*., 2025).

These observations raise important questions regarding the broader consequences of PPO activity for recombinant protein production and biochemical analyses. First, it remains unclear whether stable genetic removal of PPO activity confers similar or greater benefits compared to transient silencing approaches. Second, the extent to which PPO‐mediated crosslinking affects endogenous enzymes and their measurable activities has not been systematically investigated. Third, it is unknown whether PPO depletion improves not only protein yield but also the purity of recombinant proteins purified under nondenaturing conditions, which is essential for functional, structural, and interaction studies.

Here, we address these questions by generating and characterizing genome‐edited *N. benthamiana* lines lacking the two major *PPO* genes. We show that these *ppo* double knockout lines grow normally, exhibit reduced enzymatic browning and native protein crosslinking, preserve endogenous proteins at their predicted molecular weights, and display increased detectable enzymatic activities. Importantly, we demonstrate that PPO depletion substantially improves the yield and purity of a transiently expressed recombinant protein purified from total leaf extracts. Together, our results establish PPO knockout plants as a powerful resource for both plant science and molecular pharming applications.

## Materials and Methods

### Plant materials and growth conditions

*Nicotiana benthamiana* Domin (LAB strain) was transformed with a T‐DNA carrying Cas9 and two single guide RNAs targeting each gene (Supporting Information Table S1). Two independent CRISPR mutants were identified as homozygous by PCR and sequencing using sequencing primers listed in Table S1. The plants were maintained in a controlled growth room environment at 22°C. The plants were exposed to a 16‐h light : 8‐h dark photoperiod, under an illumination intensity of 2000 cd sr m^−2^.

### Agroinfiltration

*Agrobacterium tumefaciens* GV3101 (pMP90) was used for agroinfiltration. Agrobacterium cultures were grown overnight in LB medium supplemented with 50 μg ml^−1^ kanamycin, 10 μg ml^−1^ gentamicin, and 50 μg ml^−1^ rifampicin at 28°C with shaking at 200 rpm. For transient protein expression assays, overnight cultures of Agrobacterium strains carrying binary vectors listed in Table S2 were harvested by centrifugation. Bacterial pellets were resuspended in infiltration buffer (10 mM MgCl_2_, 10 mM MES, pH 5.7, 100 μM acetosyringone) and mixed at a 1 : 1 ratio with Agrobacterium harboring the silencing suppressor p19, with each strain adjusted to an OD₆₀₀ of 0.5. After incubation at 28°C for 1 h, bacterial suspensions were infiltrated into leaves of 4‐wk‐old *N. benthamiana* plants. Inoculated plants were maintained in a growth chamber until further use.

### Molecular cloning

To generate a plasmid encoding PPO1 flanked by FLAG and His tags, total RNA was extracted from leaves of *c*. 4‐wk‐old wild‐type *N. benthamiana* plants (FastPure Universal Plant Total RNA Isolation Kit, RC411‐01; Vazyme, Nanjing, China), and the full‐length open reading frame (ORF) of *PPO1* was amplified (Q5® Hot Start High‐Fidelity DNA Polymerase, M0493S, New England Biolabs, Hitchin, UK) by reverse transcription polymerase chain reaction. The amplified ORF was subsequently recombined into a vector containing the 35S promoter, an N‐terminal FLAG tag (Zheng *et al*., 2024), and a C‐terminal His tag using ClonExpress Ultra One Step Cloning Kit V2 (C116‐02; Vazyme), resulting in pKZ205.

### SDS‐PAGE and western blot

For total protein extraction from *N. benthamiana* leaves, samples were rapidly frozen in liquid nitrogen and ground to a fine powder with glass beads using a homogenizer. Depending on the experimental requirements, Tris‐buffered saline (TBS), phosphate‐buffered saline (PBS), or 2× gel loading buffer (GLB) was added. When total protein was extracted using TBS or PBS, 4× GLB was subsequently added for SDS‐PAGE analysis. After addition of the loading buffer, samples were denatured at 98°C for 5 min and then loaded onto 12% SDS‐PAGE gels and electrophoresed at 180 V in Invitrogen Novex vertical gel tanks. PageRuler™ Prestained Protein Ladder, 10–180 kDa (26 616; Thermo Scientific™, Waltham, MA, USA) was used as the protein ladder. Proteins were transferred to PVDF membranes using a BIO‐RAD transfer apparatus and kit (*Trans*‐Blot Turbo RTA Midi 0.45 μm LF PVDF Transfer Kit, 1704 275, Bio‐Rad Laboratories Inc., Hercules, CA, USA), following the manufacturer's instructions. Membranes were blocked in 5% skim milk in PBS‐T (PBS tablets, 524 650, Merck KGaA, Darmstadt, Germany; 0.1% Tween‐20, P1379, Merck KGaA) at room temperature for 1 h and then incubated overnight at 4°C with primary antibodies against PPO (MBS9458497; MyBioSource, San Diego, CA, USA), aldolase (ALD) (AS08294; Agrisera, Vännäs, Sweden), serine hydroxymethyltransferase (SHMT) (AS05075; Agrisera, Vännäs, Sweden), and GFP (Ab6663; Abcam, Cambridge, UK), diluted 1 : 5000 in PBS‐T. Primary antibody against RbcL (AS03037; Agrisera, Vännäs, Sweden) was diluted 1 : 10 000 in PBS‐T. For PPO detection, membranes were further incubated with goat anti‐mouse secondary antibody. Membranes were washed three times with PBS‐T for 5 min each, followed by one wash with PBS for 5 min. Signals were developed using SuperSignal™ West Femto Maximum Sensitivity Substrate (34 096; Thermo Scientific™) and detected with ImageQuant® LAS‐4000 imager (GE Healthcare Life Sciences, Little Chalfont, UK).

### Serine hydrolase activity profiling

Total proteins were extracted from wild‐type (WT) and *ppo* mutant plants using 50 mM sodium acetate (NaAc) buffer (pH 6.0), with minor modifications to the previously described procedure (Kaschani *et al*., 2009). Briefly, 25 μl of total protein extract was incubated with 0.2 μM FP‐TAMRA (Thermo Scientific™) and adjusted to a final volume of 500 μl with 50 mM NaAc (pH 6.0). The reaction mixtures were incubated for 1 h at room temperature in the dark. Proteins were then precipitated by the addition of four volumes of acetone, followed by centrifugation at 10 000*g* for 5 min at 4°C. After removal of acetone, the protein pellets were air‐dried and resuspended in 2× SDS GLB, followed by heating at 98°C for 5 min. Samples were subsequently loaded onto 12% SDS‐PAGE gels and electrophoresed at 180 V using Invitrogen Novex vertical gel tanks. PageRuler™ Prestained Protein Ladder (10–180 kDa; 26 616, Thermo Scientific™) was used as the molecular weight marker. Fluorescent signals were detected using a Typhoon FLA 9000 imaging system (GE Healthcare Life Sciences, Little Chalfont, UK) with the Cy3 scanning channel.

### Tyrosinase activity assay

Total proteins were extracted from leaves of WT and *ppo* mutant plants using PBS buffer. Tyrosinase activity was subsequently measured using the Tyrosinase Activity Assay Kit (Colorimetric) (ab252899; Abcam) according to the manufacturer's instructions. Briefly, 25 μl of total protein extract was mixed with 60 μl of Tyrosinase Assay Buffer, 10 μl of Tyrosinase Substrate, and 5 μl of Tyrosinase Probe. The reaction mixtures were incubated in a microplate reader prewarmed to 37°C, and absorbance was measured at 510 nm.

### P69B‐His purification

Total protein was extracted from agroinfiltrated leaves expressing P69B‐His using PBS. The P69B‐His protein was incubated with Ni‐NTA Agarose (30 210; Qiagen, Hilden, Germany) at 4°C for 1 h, washed three times with wash buffer (50 mM Tris–HCl, pH 7.5, 150 mM NaCl, 10 mM imidazole, 0.2% Tween‐20), and subsequently eluted using elution buffer (50 mM Tris–HCl, pH 7.5, 150 mM NaCl, 200 mM imidazole) to recover the purified protein.

### Sample preparation for Mass Spectrometry

After expressing the target protein in *N. benthamiana*, leaves were collected. After grinding in liquid nitrogen, proteins were extracted in PBS (1 g leaves in 4 ml PBS) and centrifuged to retain the input samples/supernatant (samples KZ01–KZ06, 150 μl). Part of the extract (3 ml) was applied to a first round of enrichment using Ni‐NTA Agarose in PBS. After three washes with Wash Buffer (50 mM pH 7.5 Tris–HCl, 150 mM NaCl, 10 mM imidazole, 0.2% Tween‐20), bound proteins were eluted with Elution buffer (50 mM Tris–HCl pH 7.5, 150 mM NaCl, 200 mM imidazole). The buffer was then exchanged to PBS using an Illustra™ NAP™‐5 column (17‐0853‐01, Cytiva, Marlborough, MA, USA). A 100 μl of this 1.2 ml elution was saved and precipitated with four volumes of acetone (samples KZ07–KZ12). Next, we performed a second round of 6xHis protein enrichment with the remaining samples. After washing three times with Wash Buffer, proteins were eluted with Elution Buffer and again desalted into PBS, giving samples KZ13–KZ18. All protein samples were then precipitated by addition of four volumes of acetone and incubation at −20°C for 1 h. The precipitated proteins were then collected by centrifugation. The supernatant was removed and the pellet dried on air briefly. Next, samples were taken up in 100 μl of 1× SP3 loading buffer (1% SDS, 10 mM TCEP, 40 mM CAM, 50 mM HEPES) and heated to 90°C for 5 min while shaking at 1300 rpm on a Thermocycler C (Eppendorf). The samples were then cleared by centrifugation and the protein concentration measured using the Pierce 660 nm Assay. Subsequently, we removed 15 μg of the input sample, or all of the first enrichment and 10 μg of the second enrichment, and performed tryptic digestion following the SP3 sample preparation protocol (Hughes *et al*., 2019). Tryptic peptides were finally desalted on homemade stagetips (Rappsilber *et al*., 2007).

### LC‐MS/MS settings

MS experiments were performed on an Orbitrap Fusion Lumos instrument (Thermo, Waltham, MA, USA) coupled to a Vanquish Neo ultra‐performance liquid chromatography (UPLC) system (Thermo). The UPLC was operated in the one‐column mode. The analytical column was a fused silica capillary (75 μm × 28 cm) with an integrated fritted emitter (CoAnn Technologies) packed in‐house with Kinetex 1.7 μm core shell beads (Phenomenex). The analytical column was encased by a column oven (Sonation PRSO‐V2) and attached to a nanospray flex ion source (Thermo). The column oven temperature was set to 40°C during sample loading and data acquisition. The LC was equipped with two mobile phases: solvent A (0.2% FA, 2% Acetonitrile, ACN, 97.8% H_2_O) and solvent B (0.2% FA, 80% ACN, 19.8% H_2_O). All solvents were of UPLC grade (Honeywell). Peptides were directly loaded onto the analytical column with a maximum flow rate that would not exceed the set pressure limit of 950 bar (usually *c*. 0.4–0.6 μl min^−1^). Peptides were subsequently separated on the analytical column by running a 60 min gradient of solvent A and solvent B (for details about gradient composition, see Tables S3–S7) at a flow rate of 300 nl min^−1^. The mass spectrometer was controlled by the Orbitrap Fusion Lumos Tune Application (v.4.1.4244) and operated using the Xcalibur software (v.4.7.69.37). Detailed settings for the mass spectrometer can be found in Tables S3–S7.

### Data‐processing protocol LC‐MS

RAW spectra were submitted to a closed MSFragger (v.4.1, Kong *et al*., 2017) search in Fragpipe (v.22) (Yu *et al*., 2023) using the ‘LFQ‐MBR’ workflow (label‐free quantification and match‐between‐runs; default settings were used unless otherwise stated). RAW files were listed in the ‘Input LC‐MS Files’ section and experiment set ‘by file name’. As ‘Data Type’ we kept the default ‘DDA’ (data‐dependent acquisition). The MS/MS spectra were searched against a custom database generated in Fragpipe 2025‐11–14‐decoys‐contam‐ACE_1035_NbLab360.v103.gff3.CDS.fasta.AA_plus_SOI.fasta.fas (45 850 entries including contaminants and the same number of decoys). The database comprises mainly of the *N. benthamiana* reference proteome NbLab360.v103.gff3.CDS.fasta (45 730 entries) plus the two sequences of interest (P69B and EPI1). MSFragger searches allowed oxidation of methionine residues (16 Da; 3) and acetylation of the protein N‐terminus (42 Da; 1), as variable modifications (first value in brackets refers to the molecular weight of the modification, second value to the maximum number of occurrences per peptide). A maximum of three variable modifications and a maximum of five combinations were allowed globally. Carbamidomethylation on cysteine (57) was selected as static modification. Enzyme specificity was set to ‘Trypsin’. The initial precursor and fragment mass tolerance was kept at ±20 ppm. Mass calibration and parameter optimization was selected. Validation of peptide spectrum matches was done by running MSBooster (Yang 2023) using DIA‐NN for RT and spectra prediction. Peptide spectra matches were validated using percolator with a minimum probability setting of 0.5. Protein inference was performed using ProteinProphet (part of Philosopher v.5.1.1). The final reported protein False Discovery Rate (FDR) was 0.01 (based on target‐decoy approach). Protein quantification was performed with IonQuant (v.1.10.27). MaxLFQ (min ions = 1), MBR (FDR 0.01), and normalization of intensities across runs was selected. Unique and razor peptides were allowed. Advanced options were kept at default. Further analysis and filtering of the results were done in Perseus v.1.6.10.0 (Tyanova *et al*., 2016). Comparison of protein group quantities (relative quantification) between different MS runs is based solely on the LFQs as calculated by IonQuant, MaxLFQ algorithm.

### Statistical analysis

All statistical analyses were performed in GraphPad Prism (v.11.0.0). Comparisons between WT and mutant lines were made using one‐way ANOVA (ordinary, Welch's, or Kruskal–Wallis, depending on whether assumptions of normality and homoscedasticity were met), with *post hoc* comparisons vs WT conducted using Dunnett's, Dunnett's T3, or Dunn's test as appropriate. For experiments involving multiple factors, two‐way ANOVA was performed with Geisser–Greenhouse correction applied where relevant, and *post hoc* comparisons vs WT used Dunnett's or Šídák‐adjusted tests. Data are presented as mean ± SEM, and *P* < 0.05 was considered statistically significant.

## Results

### Generation and characterization of *ppo* double knockout lines

**Fig. 1:**
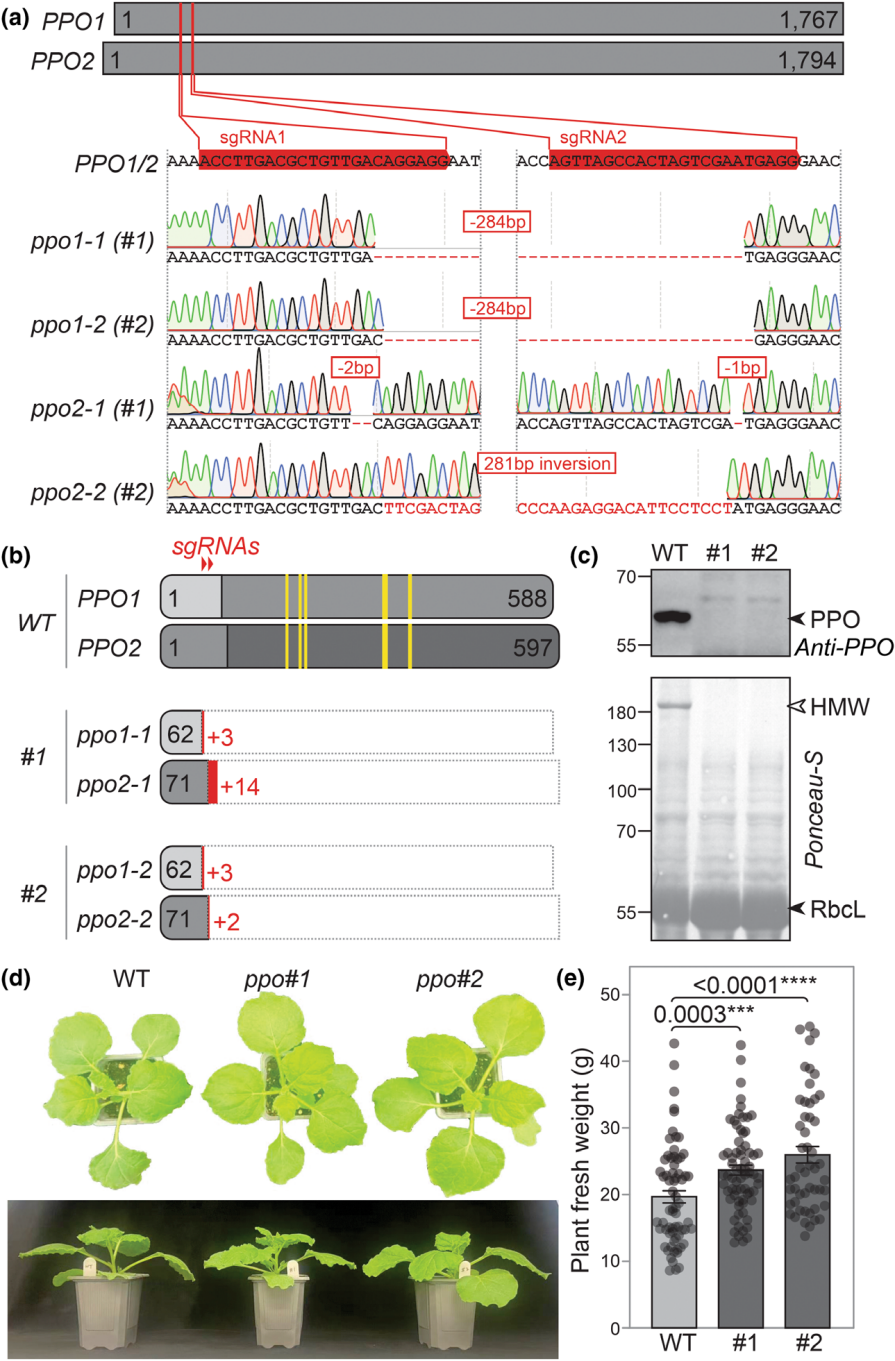
Characteristics of *ppo* double knockout mutant lines. (a) CRISPR/Cas9‐induced nucleotide alterations in polyphenol oxidases (*PPO*) genes of *Nicotiana benthamiana*. The 5′ region of the open reading frames of *PPO1* and *PPO2* was targeted by two single guide RNAs (sgRNAs, red). The sgRNA target locus was sequenced in homozygous lines #1 and #2 carrying alleles *ppo1‐1* and *ppo2‐1* in line #1 or alleles *ppo1‐2* and *ppo2‐2* in line #2, respectively. Shown are the alignments with wild‐type (WT) *PPO1* and *PPO2*, which are identical in the shown regions. (b) Predicted proteins encoded by the mutant *ppo* alleles. The length of the open reading frame including the nonsense sequence (red) is indicated in amino acid residues. The prodomain (light grey) and catalytic residues (yellow) are highlighted. (c) The PPO protein is absent in leaf extracts of *ppo* mutants. Leaf extracts from 4‐wk‐old plants were analyzed by Ponceau‐S staining and western blot analysis using the anti‐PPO antibody. (d) Top image of 28‐d‐old WT and *ppo* mutant plants. (e) Mutant *ppo* plants are slightly larger than WT plants. The fresh weight of the plants aboveground was measured in four sets of 30–31‐d‐old plants. Error bars represent SE of *n* > 50 replicates generated in four batches of plants. Statistical significance was assessed using ANOVA. ***, *P* < 0.001, ****, *P* < 0.0001.

The 300 bp fragment that we previously used to silence *PPO* (Mahadevan *et al*., 2025) targets two highly homologous *PPO* genes: *PPO1* (NbL17g16540) and *PPO2* (NbL10g18390). According to LAB360 and NbenBase genome annotations (Kurotani *et al*., 2023; Ranawaka *et al*., 2023), *N. benthamiana* has seven *PPO* genes (Fig. S1a). However, only *PPO1* is significantly expressed in leaves, and its expression increases upon agroinfiltration (Grosse‐Holz *et al*., 2018, Fig. S1b). To generate the *ppo* knockout lines, we designed two single guide RNAs (sgRNAs) targeting these *PPO* genes, and we selected two independent homozygous lines carrying disrupted open reading frames for both genes. Line #1 carries a 284 bp deletion in *PPO1* (allele *ppo1‐1*), and 2 bp and 1 bp deletions in *PPO2* (allele *ppo2‐1*), whereas line #2 carries a different 284 bp deletion in *PPO1* (allele *ppo1‐2*) and a 281 bp inversion in PPO2 (allele *ppo2‐2*) (Fig. 1a). Consequently, both *PPO1* and *PPO2* were disrupted early in the open reading frames before encoding the catalytic residues (Fig. 1b), indicating that these lines are double null mutants for both *PPO* genes. Western blot analysis of leaf extracts with anti‐PPO antibodies indeed confirmed that both lines lack the PPO protein (Fig. 1c). A Ponceau‐S stain of the corresponding membrane shows equal loading but also displays a 180 kDa high molecular weight (HMW) signal for WT plants that is absent from *ppo* mutant lines (Fig. 1c).

Both *ppo* mutants grew and developed normally (Fig. 1d), but the *ppo* mutants seemed to grow slightly larger. The *ppo* mutants indeed accumulate 20–32% more fresh weight (Fig. 1e), which is a great benefit to protein production yields.

### PPO depletion reduces enzymatic browning and native protein crosslinking

**Fig. 2:**
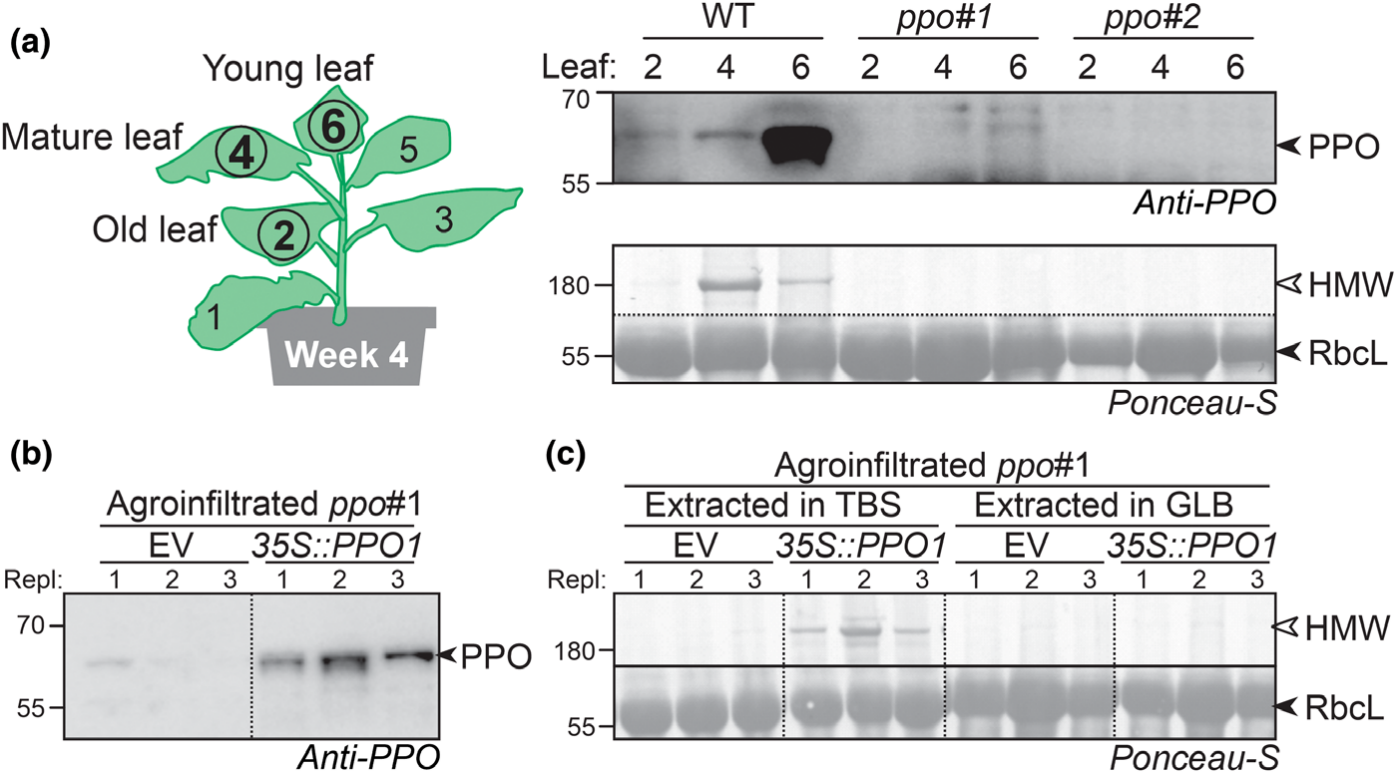
Polyphenol oxidases (PPOs) are responsible for crosslinking. (a) Native crosslinking into high molecular weight (HMW) complexes in mature leaves is absent in *ppo* mutants of *Nicotiana benthamiana*. Leaf extracts from leaf discs of young, mature, and old leaves were generated in Tris‐buffered saline (TBS) and analyzed by Ponceau‐S staining and western blot analysis using the anti‐PPO antibody. (b) Transient expression of *35S::PPO1* triggers PPO accumulation in *ppo* mutant. Leaves of the *ppo* mutant #1 were agroinfiltrated, and samples were analyzed 4 d later by western blot using the anti‐PPO antibody. (c) Native crosslinking into the HMW complex occurs in extracts of PPO1 expressing leaves in TBS, but not in gel‐loading buffer (GLB). RbcL, ribulose‐1,5‐bisphosphate carboxylase/oxygenase.

PPO accumulation clearly correlates with leaf age, with most PPO protein accumulating in young, expanding leaves and being absent in both *ppo* mutants (Fig. 2a). However, the 180 kDa HMW signal is strongest in mature leaves (Fig. 2a), which are most often selected for agroinfiltration for their high expression and ease of infiltration.

To confirm the role of PPO in native crosslinking, we transiently overexpressed PPO1 and the empty vector (EV) in the *ppo* mutant by agroinfiltration. Western blot analysis confirmed that PPO accumulated in leaves agroinfiltrated with *35S::PPO1* and not in the EV control (Fig. 2b), but PPO levels are not much higher than endogenous PPO levels, despite the use of the strong CaMV 35S promoter. Ponceau‐S staining revealed that the 180 kDa HMW signal accumulated upon PPO expression when extracts were generated in TBS, but not upon extraction in GLB containing reducing agent dithiothreitol and sodium dodecyl sulfate (SDS) (Fig. 2c), confirming that PPO‐induced protein crosslinking occurs during extraction and that PPO inactivation can prevent this.

### Transient protein expression is not altered in *ppo* mutants

We previously noticed that transient expression of GFP might be higher in *TRV::PPO* plants (Mahadevan *et al*., 2025). In our *ppo* mutants, however, fluorescence is not increased upon transient GFP expression (Fig. S2a), and similar levels of GFP protein accumulated in the *ppo* mutants (Fig. S2b), indicating that transient expression of GFP is not significantly different in *ppo* mutants compared with WT plants.

### PPO depletion preserves endogenous proteins at predicted molecular weights

**Fig. 3:**
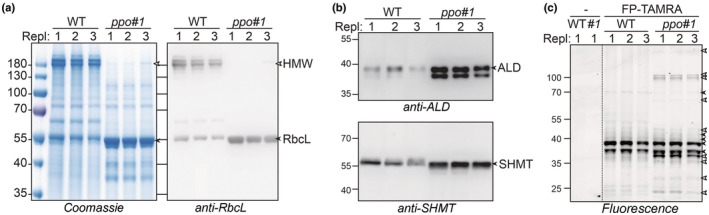
Polyphenol oxidases (PPOs) depletion preserves endogenous proteins at their predicted molecular weights and enhances detectable enzymatic activities. (a) Less RbcL crosslinking in *ppo* mutant of *Nicotiana benthamiana*. Leaf extracts were incubated for 1 h, separated on protein gels, stained with Coomassie or analyzed by Western blot with the anti‐RbcL antibody on *n* = 3 replicates. (b) Increased levels of endogenous enzymes at their predicted molecular weight. Proteomes used in (a) were probed with antibodies against fructose‐bisphosphate aldolase (ALD) and serine hydroxymethyltransferase (SHMT). (c) More activity‐based signals detected in extracts of *ppo* mutants. Leaf extracts were labeled with FP‐TAMRA, which covalently binds active serine hydrolases for 60 min, separated on protein gels and scanned for fluorescence in *n* = 3 replicates. HMW, high molecular weight; RbcL, ribulose‐1,5‐bisphosphate carboxylase/oxygenase; WT, wild‐type.

To investigate how PPO depletion affects the integrity of endogenous proteins under nondenaturing extraction conditions, we incubated total leaf extracts from WT and *ppo* mutant plants in PBS for 1 h and analyzed protein migration by SDS‐PAGE. Coomassie staining revealed that incubation of WT extracts resulted in a pronounced loss of proteins migrating at their predicted molecular weights, accompanied by the appearance of a prominent HMW signal *c*. 180–200 kDa (Fig. 3a). By contrast, extracts from *ppo* mutants retained substantially stronger signals at their expected molecular weights and displayed a marked reduction in HMW material (Fig. 3a).

Immunoblot analysis using an antibody against RbcL demonstrated that the HMW signal observed in WT extracts contains RbcL, whereas RbcL in *ppo* mutant extracts predominantly migrated at its predicted molecular weight of *c*. 55 kDa (Fig. 3a). Quantification revealed that the RbcL signal at 55 kDa was *c*. twofold higher in *ppo* mutant extracts compared with WT, indicating that PPO depletion preserves RbcL in its native, noncrosslinked form.

To assess whether this effect extends beyond RbcL, we probed the same extracts with antibodies against fructose‐bisphosphate ALD and SHMT, two abundant metabolic enzymes with predicted molecular weights of 41.9 kDa and 54.8 kDa, respectively. Both ALD and SHMT displayed significantly stronger signals at their predicted molecular weights in *ppo* mutant extracts compared to WT, with increases of 2.32‐fold and 1.48‐fold, respectively (Fig. 3b). These results indicate that PPO‐mediated crosslinking broadly affects multiple endogenous proteins during extraction and that genetic removal of PPO preserves a wider range of proteins in their native molecular forms.

Because preservation of protein integrity is expected to improve the detection of enzymatic activities, we next performed activity‐based profiling of serine hydrolases using the fluorescent probe FP‐TAMRA. FP‐TAMRA is a fluorescent fluoro‐phosphonate probe that reacts with hyperreactive serine residues in the active site of serine hydrolases in an activity‐dependent manner (Liu *et al*., 1999). Labeling of extracts from *ppo* mutant plants revealed nine additional fluorescent signals compared with WT extracts (Fig. 3c). These additional signals indicate that more active serine hydrolases are detectable in the absence of PPO‐mediated crosslinking, suggesting that PPO activity limits the measurable activity landscape of endogenous enzymes in leaf extracts.

### PPO‐dependent tyrosinase activity underlies native protein crosslinking

**Fig. 4:**
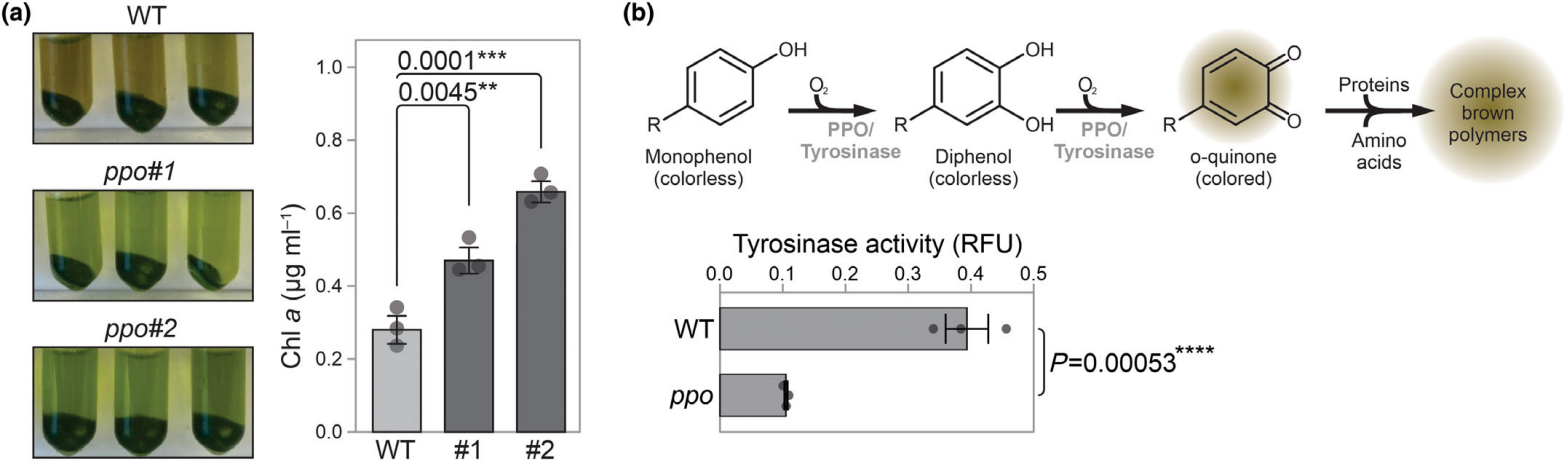
Polyphenol oxidases (PPOs) depletion reduces enzymatic browning and tyrosinase activity in *Nicotiana benthamiana* leaf extracts. (a) Leaf extracts of *ppo* mutants of *N. benthamiana* are less brown and greener and contain higher Chl *a* levels. Left: leaf extracts were incubated in Tris‐buffered saline (TBS) for 2 min and centrifuged before the image was taken. Right: leaf extracts were incubated with 80% acetone and the concentrations of Chl *a* were measured by absorbance spectroscopy. Error bars represent SE of *n* = 3 biological replicates. *P*‐values were calculated with ANOVA. (b) Phenol oxidation by PPO/Tyrosinase can cause protein polymers (upper panel). Reduced tyrosinase activity in extracts of *ppo* mutants (lower panel). Leaf extracts were assayed for tyrosinase activity in phosphate‐buffered saline. Error bars represent SE of *n* = 3 replicates. *P*‐value was calculated with Student's *t*‐test. WT, wild‐type. ****, *P* < 0.0001.

Because the leaf extracts of *ppo* mutants not only appeared less brown but also much greener (Fig. 4a), we quantified Chl levels in acetone extracts by absorbance. These measurements indicate a significant 68–135% increase of Chl‐*a* in *ppo* mutants (Fig. 4a), suggesting that Chl is oxidized by PPO in leaf extracts.

Polyphenol oxidases can promote protein crosslinking through two related mechanisms: the oxidation of phenolic compounds released during tissue disruption and the direct oxidation of solvent‐exposed tyrosine residues on proteins via tyrosinase activity. Both processes generate reactive quinones that can form covalent bonds with nucleophilic amino acid side chains, resulting in protein polymerization (Fig. 4b).

To determine whether PPO‐dependent tyrosinase activity contributes to the observed native protein crosslinking, we measured tyrosinase activity in total leaf extracts from wild‐type and *ppo* mutant plants. Extracts from *ppo* mutants displayed a threefold reduction in tyrosinase activity compared to wild‐type (Fig. 4b). This reduction is consistent with the absence of PPO protein in the mutant lines and indicates that PPO is the major contributor to tyrosinase activity in leaf extracts under the tested conditions.

These results support a model in which PPO‐mediated oxidation reactions, including tyrosine oxidation, drive the formation of HMW protein complexes during extraction. The strong reduction of both tyrosinase activity and protein crosslinking in *ppo* mutants demonstrates that genetic removal of PPO effectively suppresses these processes.

### Recombinant protein purification yield and purity are improved in *ppo* mutants

Native protein crosslinking during extraction is expected to reduce the recovery of recombinant proteins by trapping them in insoluble or HMW complexes. To test whether PPO depletion improves recombinant protein purification, we transiently expressed a His‐tagged version of the tomato subtilase P69B in wild‐type and *ppo* mutant plants. P69B is a defence‐related subtilase of tomato that we study as a target for various pathogen‐secreted proteins (Homma *et al*., 2023). Immunoblot analysis of total leaf extracts revealed comparable levels of P69B‐His accumulation in both genotypes, indicating that PPO depletion does not affect transient expression of this recombinant protein (Fig. S3).

**Fig. 5:**
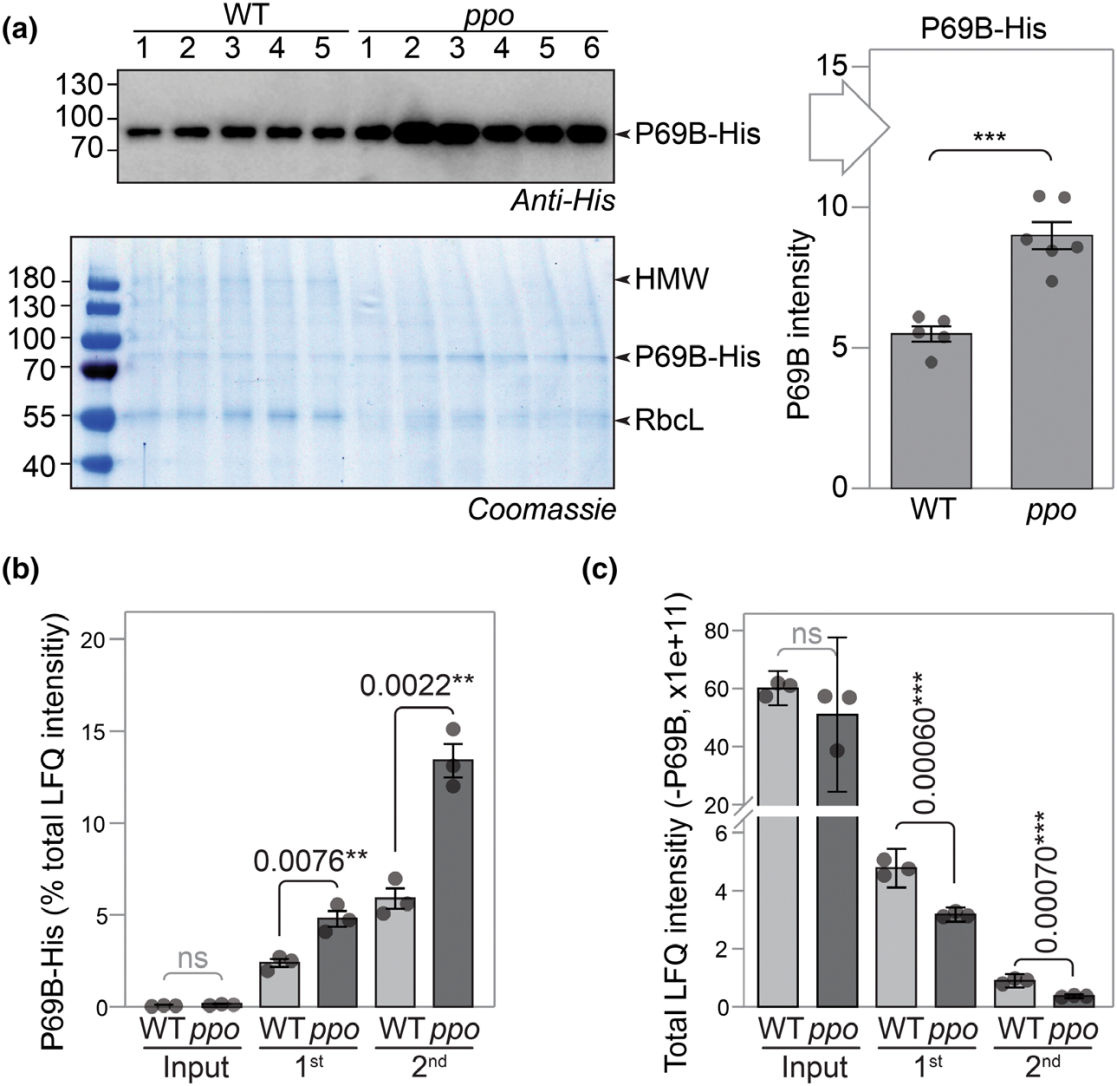
Polyphenol oxidases (PPO) depletion improves the yield and purity of a recombinant protein purified from *Nicotiana benthamiana*. (a) More P69B‐His was purified from the *ppo* mutant of *N. benthamiana*. Leaf total protein extracts of wild‐type (WT) and *ppo* mutant line #1 transiently expressing P69B‐His at 3 dpi were passed over Ni‐NTA beads in *n* = 5–6 replicates. Beads were washed, and the imidazole‐eluted fraction samples were analyzed by western blot using the anti‐His antibody and Coomassie staining. Western blot signals were quantified and plotted. Error bars represent SE of *n* = 5 replicates. *P*‐value: Student's *t*‐test, <0.001 (***). (b) Increased purity of P69B from the *ppo* mutant. P69B‐His was purified twice over Ni‐NTA from extracts of leaves transiently expressing P69B and input and eluate samples were analyzed by MS for *n* = 3 replicates. Ion intensities of P69B‐His determined by label‐free quantification (LFQ) were calculated as a percentage of the total LFQ intensity. Error bars represent SE of *n* = 3 replicates. *P*‐values: Student's *t*‐test. (c) Reduced intensity of contaminant proteins from *ppo* mutant. Total LFQ intensities without P69B‐His are shown for samples described in (b). Error bars represent SE of *n* = 3 replicates. *P*‐values: Student's *t*‐test. HMW, high molecular weight; RbcL, ribulose‐1,5‐bisphosphate carboxylase/oxygenase; WT, wild‐type.

Following extraction under nondenaturing conditions, P69B‐His was purified using Ni–NTA affinity chromatography. Although similar amounts of P69B‐His were captured on the affinity resin from WT and *ppo* mutant extracts, the amount of P69B‐His recovered in the eluate was significantly higher when purified from *ppo* mutant plants (Fig. 5a). Coomassie staining revealed the HMW signal to be present in the eluate of WT plants, but much less in that of *ppo* mutant plants (Fig. 5a), indicating that eluates from *ppo* plants are much cleaner compared to those from WT plants.

To further quantify purification efficiency and purity, we performed mass spectrometry analysis on independently purified P69B‐His samples, including a second round of Ni–NTA enrichment. The purity of P69B‐His was more than twofold higher from extracts of *ppo* mutants after both purifications, reaching 14% of the total ion intensities (Fig. 5b). The increased purity corresponded with significantly lower ion intensities of protein contaminants in the *ppo* mutant (Fig. 5c).

Together, these results demonstrate that PPO depletion substantially improves both the yield and purity of recombinant proteins purified from *N. benthamiana* leaf extracts, without altering their expression levels.

## Discussion

### PPO‐mediated crosslinking represents a bottleneck in plant protein extraction

This study demonstrates that PPOs impose a substantial and previously underappreciated constraint on protein integrity during extraction from *N. benthamiana* leaves. Genetic disruption of the two major leaf‐expressed *PPO* genes resulted in viable *ppo* double knockout plants without obvious developmental defects and with slightly increased biomass (Fig. 1d,e), indicating that PPO depletion is well tolerated under standard growth conditions. Despite this mild whole‐plant phenotype, PPO removal had profound consequences for protein integrity during extraction.

Although enzymatic browning has long been recognized as a nuisance in plant protein purification, our results show that PPO activity has broader consequences by driving extensive native protein crosslinking that alters protein migration, reduces detectable enzymatic activities, and compromises recombinant protein recovery (Figs 2, 3, 4, 5). In WT plants, extraction under nondenaturing conditions resulted in rapid browning and the formation of HMW protein material (Figs 2a and 3a), illustrating how quickly PPO‐mediated reactions are triggered upon tissue disruption.

At the protein level, PPO‐mediated oxidation caused abundant stromal enzymes such as RbcL to shift into HMW complexes in WT extracts, whereas RbcL remained predominantly at its predicted molecular weight in *ppo* mutant extracts (Fig. 3a). This effect was not restricted to RbcL but extended to other endogenous enzymes, including fructose‐bisphosphate aldolase and serine hydroxymethyltransferase (Fig. 3b), indicating that PPO‐mediated crosslinking broadly affects the leaf proteome.

### Consequences of PPO depletion for endogenous enzyme activity profiling

The preservation of proteins at their predicted molecular weights in *ppo* mutants was accompanied by an increase in detectable enzymatic activities. Activity‐based protein profiling revealed a greater number of active serine hydrolases in extracts from *ppo* mutants compared with WT (Fig. 3c), indicating that PPO activity restricts the measurable activity landscape of endogenous enzymes. Because activity‐based probes require access to intact and catalytically competent active sites, these results suggest that PPO‐mediated oxidation and crosslinking mask or inactivate enzyme activities in WT extracts. Taken together, our results indicate that *ppo* mutants will offer significant advantages in studies on native proteomes, including protein activities and protein–protein interactions.

Mechanistically, PPOs contribute to the oxidative capacity of leaf extracts through both phenolic substrates and oxidation of solvent‐exposed tyrosine residues. Consistent with this, extracts from *ppo* mutants displayed strongly reduced tyrosinase activity (Fig. 4b). Together with the conceptual model shown in Fig. 4b, these data support a mechanism in which PPO activity increases the oxidative potential for protein browning and polymer formation during extraction, thereby indirectly limiting enzyme activity detection.

These observations have broader implications for functional proteomics in plants. Many studies aim to characterize enzyme activities under near‐native conditions, yet the contribution of PPO‐mediated reactions to activity loss has not been systematically considered. Our results indicate that PPO depletion provides a straightforward genetic strategy to improve the reliability of activity‐based profiling and biochemical assays performed on leaf extracts.

### Implications for recombinant protein yield and purity in molecular pharming

Recombinant protein production in *N. benthamiana* relies heavily on downstream purification from total leaf extracts, where native crosslinking can trap proteins in insoluble or heterogeneous assemblies. Importantly, PPO depletion did not increase transient expression levels, as GFP accumulation and fluorescence were comparable between WT and *ppo* mutant plants (Fig. S2a,b). This demonstrates that differences observed during purification cannot be attributed to altered expression efficiency.

Instead, PPO depletion substantially improved downstream recovery. The significant increase in P69B‐His recovery from *ppo* mutant extracts (Fig. 5a) demonstrates that PPO‐mediated crosslinking constitutes a major bottleneck during purification. Moreover, mass spectrometry–based analysis revealed a marked increase in the purity of recombinant protein preparations derived from *ppo* mutants, accompanied by a corresponding reduction in contaminating plant proteins (Fig. 5b,c).

These findings extend previous observations made using transient *PPO* silencing approaches and demonstrate that stable genome‐edited *PPO* knockout lines provide consistent and robust benefits for protein purification (Mahadevan *et al*., 2025). Unlike chemical inhibitors or transient silencing strategies, genetic removal of *PPO* eliminates the need for additional treatments and avoids variability associated with incomplete suppression.

Our findings are consistent with a recent report that knocking out the same two *PPOs* from *N. benthamiana* improves recombinant protein purification (Diao *et al*., 2026). Similar to our observations, Diao and colleagues found no increased transient expression of GFP in *ppo* mutants and reported significant improvements in yield and purity of recombinant proteins upon purification.

### Comparison with alternative strategies to mitigate extraction‐induced artifacts

Various strategies have been employed to mitigate enzymatic browning and protein degradation during plant protein extraction, including the use of reducing agents, polyvinylpolypyrrolidone, protease inhibitors, and rapid denaturation. Although such approaches can partially suppress browning, they are often incompatible with experiments requiring native protein structure and activity. By contrast, genetic removal of *PPO* intrinsically suppresses a major source of oxidation‐driven crosslinking at its origin, as evidenced by reduced browning, preserved protein migration, and improved enzyme activity detection (Figs 2, 3, 4).

Recent advances in genome editing, VIGS, and RNAi technology have facilitated the generation of plant lines optimized for transient expression and protein production, such as plants deficient in immune receptors (Dodds *et al*., 2025) or proteases (Beritza *et al*., 2024b) and alkaloids (Vollheyde *et al*., 2023). The *ppo* mutants described here complement these approaches by addressing a distinct but equally critical bottleneck at the extraction and purification stage.

### Future applications

Polyphenol oxidases have been implicated in plant defense and stress responses (Zhang & Sun, 2021; Zhang, 2023), and their removal may influence susceptibility to pests or pathogens under certain conditions (Felton *et al*., 1989; Constabel *et al*., 1995; Thipyapong *et al*., 2004; Bhonwong *et al*., 2009). However, the absence of obvious developmental defects and the slightly increased biomass observed in *ppo* mutants suggest that PPO deletion is well tolerated under controlled growth conditions (Fig. 1d,e).

Future studies could explore the combination of *PPO* knockout lines with other genetic modifications designed to enhance transient expression, reduce proteolysis, or suppress immune responses. Such combinatorial approaches may further optimize *N. benthamiana* as a versatile chassis for plant science and molecular pharming. In addition, extending PPO depletion strategies to other plant species used for protein production could broaden the applicability of these findings.

### Conclusion

We have introduced two independent *ppo* double mutant lines that lack the PPO protein and have less native crosslinking and browning in leaf extracts. With reduced native crosslinking, leaf extracts of *ppo* mutants maintain native proteins at their predicted molecular weight and facilitate the detection of more enzyme activities. Transient GFP expression is not affected in *ppo* mutants, but the purification yield and purity of recombinant proteins are significantly improved. These data indicate that these *ppo* lines will be instrumental for both plant science and the molecular pharming industry by enabling the production and study of purified proteins in their native state *in planta*.

## Competing interests

None declared.

## Author contributions

RALVdH conceived the project; KZ performed the experiments; FK and MK did proteomic analysis; ECW did bioinformatic analysis and statistics; RALVdH and KZ wrote the manuscript with input from all authors.

## Disclaimer

The New Phytologist Foundation remains neutral with regard to jurisdictional claims in maps and in any institutional affiliations.

## Supporting information


**Fig. S1** *PPO* genes in *Nicotiana benthamiana*.
**Fig. S2** Transient protein expression is unaltered in *ppo* mutants.
**Fig. S3** Input samples for P69B‐His purification.
**Table S1** Used oligonucleotides.
**Table S2** Used plasmids.
**Table S3** Samples used for proteomics.
**Table S4** LC settings.
**Table S5** MS settings.
**Table S6** MSFragger search.
**Table S7** MaxQuant search.
Please note: Wiley is not responsible for the content or functionality of any Supporting Information supplied by the authors. Any queries (other than missing material) should be directed to the *New Phytologist* Central Office.

## Data Availability

The mass spectrometry proteomics data for the digestions have been deposited to the ProteomeXchange Consortium via the PRIDE (Vizcaíno *et al*., 2016) partner repository (https://www.ebi.ac.uk/pride/archive/) with the dataset identifier PXD071885.
