# Supplementary material for: Polyphenol oxidase depletion in Nicotiana benthamiana enhances recombinant protein purification and preserves native protein integrity
Source: New Phytol. 2026 May 24;251(3):1283–93. doi: 10.1111/nph.71262 (PMC13326509; doi:10.1111/nph.71262)
Supplement: Supplementary file 1 — Fig. S1 PPO genes in Nicotiana benthamiana. Fig. S2 Transient protein expression is unaltered in ppo mutants. Fig. S3 Input samples for P69B‐His purification. Table S1 Used oligonucleotides. Table S2 Used plasmids. Table S3 Samples used for proteomics. Table S4 LC settings. Table S5 MS settings. Table S6 MSFragger search. Table S7 MaxQuant search. Please note: Wiley is not responsible for the content or functionality of any Supporting Information supplied by the authors. Any queries (other than missing material) should be directed to the New Phytologist Central Office. [file NPH-251-1283-s001.pdf]

New Phytologist Supporting Information

Article title: *Polyphenol oxidase depletion in Nicotiana benthamiana enhances recombinant protein purification and preserves native protein integrity.*

Authors: Kaijie Zheng, Farnusch Kaschani, Emma C. Watts, Markus Kaiser and Renier A. L. van der Hoorn

Article acceptance date: 9 April 2026

SUPPLEMENTAL FIGURES

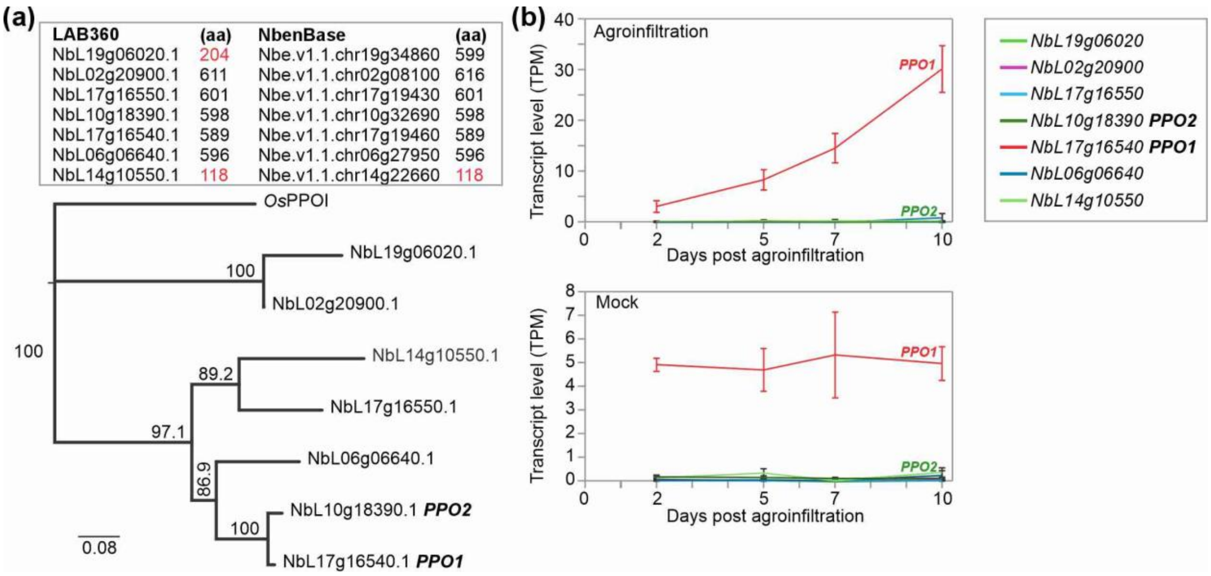

Fig. S1 *PPO* genes in *Nicotiana benthamiana*

(a) Upper panel: accession numbers of seven *PPO* genes from the LAB360 and NbenBase and the lengths of the proteins predicted from the open reading frames, showing a discrepancy only for the top listed gene. Lower panel: phylogeny of *PPO* genes. The tree was made with the Jukes–Cantor genetic distance model and the Neighbor-Joining method, with *O. sativa* PPOI as the outgroup. Branch support was assessed using 1000 bootstrap replicates. The analysis was performed in Geneious. **(b)** Transcript levels of the *PPO* genes in Mock and agroinfiltrated leaves in transcripts per million (TPM). Data were extracted from Grosse-Holz et al., 2018.

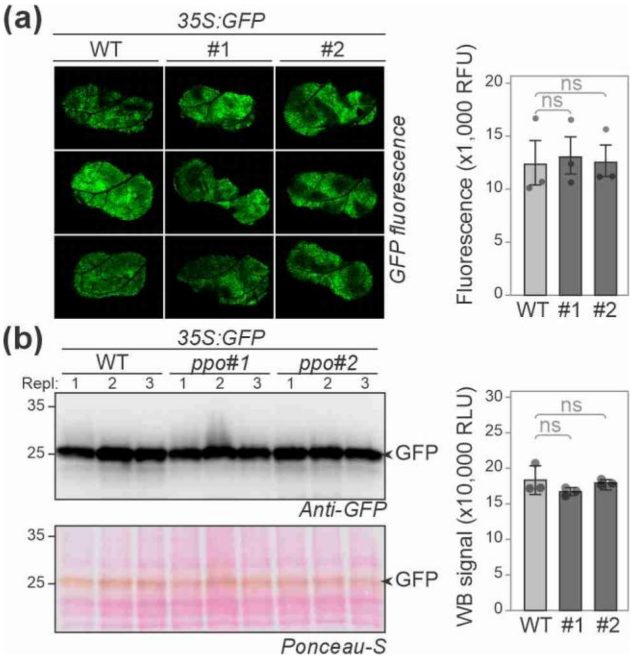

Fig. S2 Transient protein expression is unaltered in *ppo* mutants.

(a) Similar levels of GFP fluorescence upon transient expression in *ppo* mutant. Left: leaves were agroinfiltrated to transiently express *35S::GFP* and the fluorescence was imaged three days later (3dpi). Right: quantification of GFP fluorescence over three different plants. Error bars represent SE. P-values were calculated with ANOVA and found non-significant (ns, $p>0.05$). **(b)** Similar levels of GFP accumulation upon transient expression in *ppo* mutant. Left: leaf extracts generated from leaves transiently expressing GFP at 3dpi were analysed by anti-GFP western blot in n=3 biological replicates. Right: quantification of western signals. Error bars represent SE. P-values were calculated with ANOVA and found non-significant (ns, $p>0.05$).

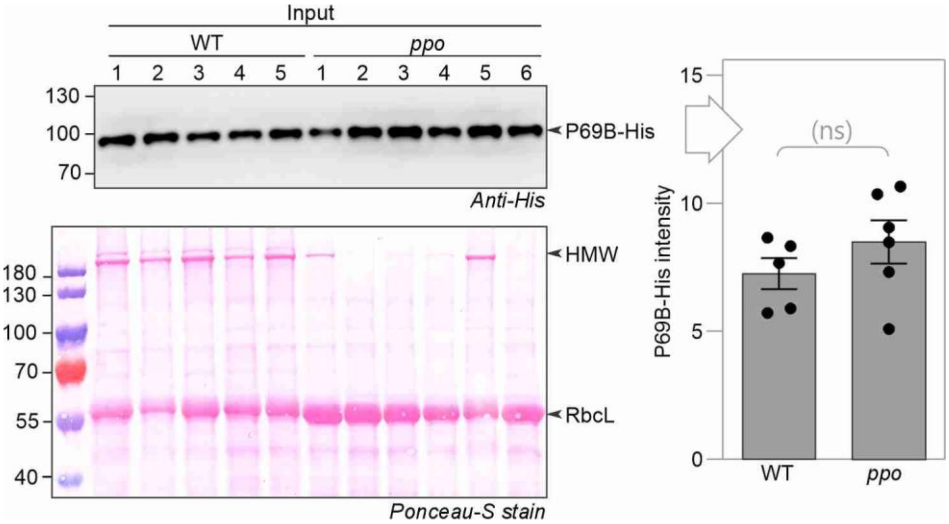

Fig. S3 Input samples for P69B-His purification.

Leaf total protein extracts of WT and *ppo* mutant line #1 transiently expressing P69B-His at 3dpi were analysed by western blot using the anti-His antibody and Ponceau-S staining. Western blot signals were quantified and plotted on the right. Error bars represent SE. P-values were determined with Student's t-test and are non-significant (ns).

SUPPLEMENTAL TABLES

Table S1 Used oligonucleotides

Name	Sequence (5'-3')
PPO1-F1	GATGACGACGATAAGCTTAAGATGGCTTCTTCTTCTACTTTACCTTTATG
PPO1-R1	ATGGTGATGGTGATGTCTAGAACAATCGACAAGCTTAATCTCAACAC
sgRNA1	ACCTTGACGCTGTTGACAGGAGG
sgRNA2	AGTTAGCCACTAGTCGAATGAGG
PPO1-seq-F1	CAGCAATGGCTTCTTCTTCTACTTT
PPO1-seq-R1	CTTGTAGCTCTTTGCCACCA
PPO2-seq-F2	CCAGCACCAACTCATCTTTCT
PPO2-seq-R1	GGTGACTTTGGTTACGTCTTGC

Table S2 Used plasmids

Name	Description	Reference
pFH20	Binary vector 35S:NtPR1a-P69B-His	Homma et al., 2023
pKZ165	Binary vector 35S:nlsGFP	This work
pKZ205	Binary vector 35S:FLAG-PPO1-2xHis	This work
pJK001c	Empty binary vector	Paulus et al., 2020
P19	Binary 35S::P19	Van der Hoorn et al., 2003

Supplemental references:

Paulus JK, Kourelis J, Ramasubramanian S, Homma F, Godson A, Hörger AC, Hong TN, Krahn D, Ossorio Carballo L, Wang S, Win J, Smoker M, Kamoun S, Dong S, van der Hoorn RAL. (2020) Extracellular proteolytic cascade in tomato activates immune protease Rcr3. *Proc Natl Acad Sci USA.* **117**: 17409-17417.

Van Der Hoorn RAL, Rivas S, Wulff BB, Jones JDG, Joosten MHAJ. (2003) Rapid migration in gel filtration of the Cf-4 and Cf-9 resistance proteins is an intrinsic property of Cf proteins and not because of their association with high-molecular-weight proteins. *Plant J.* **35**: 305-315.

Table S3 Samples used for proteomics

ACE ID	Alternate ID	Treatment/ experimental setup
ACE_1035_KZ01	WT-Input-1	Proteins were extracted by B#_A.
ACE_1035_KZ02	WT-Input-3	
ACE_1035_KZ03	WT-Input-4	
ACE_1035_KZ04	ppo-Input-2	
ACE_1035_KZ05	ppo-Input-3	
ACE_1035_KZ06	ppo-Input-4	
ACE_1035_KZ07	WT-1stP-1	Proteins were extracted by B#_A. Purified by B#_B. Then changed the buffer by column. Precipitate proteins using acetone.
ACE_1035_KZ08	WT-1stP-3	
ACE_1035_KZ09	WT-1stP-4	
ACE_1035_KZ10	ppo-1stP-2	
ACE_1035_KZ11	ppo-1stP-3	
ACE_1035_KZ12	ppo-1stP-4	
ACE_1035_KZ13	WT-2ndP-1	Proteins were extracted by B#_A. Purified by B#_B. Then changed the buffer by column. And purified by B#_B changed the buffer by column again. Precipitate proteins using acetone.
ACE_1035_KZ14	WT-2ndP-3	
ACE_1035_KZ15	WT-2ndP-4	
ACE_1035_KZ16	ppo-2ndP-2	
ACE_1035_KZ17	ppo-2ndP-3	
ACE_1035_KZ18	ppo-2ndP-4	

B#_A	PBS, https://www.sigmaaldrich.com/GB/en/product/mm/524650
B#_B	Ni-NTA Agarose, https://www.qiagen.com/us/products/discovery-and-translational-research/protein-purification/tagged-protein-expression-purification-detection/ni-nta-agarose

Table S4 LC setting

<table>
<tr><td>MS device</td><td>Orbitrap Fusion Lumos</td></tr>
<tr><td>LC device</td><td>Vanquish Neo</td></tr>
<tr><td>ion source</td><td>Thermo Nanospray Flex</td></tr>
<tr><td>Analytical column</td><td>Self-packed fused silica capillary with an integrated sintered frit; CoAnn Technologies ICT36007515F-50-5</td></tr>
<tr><td>column diameter</td><td>Length (L_C) = 28 cm; ID = 75µm; OD = 360 µm; emitter 15 µm</td></tr>
<tr><td>stationary phase</td><td>Phenomenex Kinetex C18-XB core shell</td></tr>
<tr><td>particle diameter (d_p)</td><td>1.7 µm (core shell)</td></tr>
<tr><td>Pore size</td><td>100 Å</td></tr>
<tr><td>Column ID</td><td>AC170</td></tr>
<tr><td>Column oven</td><td>Sonation column oven PRSO-V2</td></tr>
<tr><td>Column oven temp.</td><td>40°C</td></tr>
<tr><td>solvents</td><td>A: 0.2% FA, 2% ACN, 98% H_2O
B: 0.2% FA, 80% ACN, 20 % H_2O</td></tr>
<tr><td>gradient</td><td>
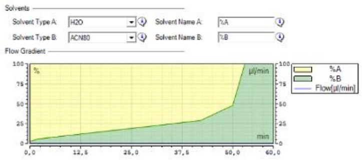

<table>
<tr><th>No</th><th>Time</th><th>Duration [min]</th><th>Flow [µl/min]</th><th>%B</th><th>Volume [µl]</th><th>No. of Column Volumes</th></tr>
<tr><td>1</td><td>0,000</td><td colspan="5">Run</td></tr>
<tr><td>2</td><td>0,000</td><td>0,000</td><td>0,300</td><td>3,0</td><td>0,00</td><td>0,00</td></tr>
<tr><td>3</td><td>2,000</td><td>2,000</td><td>0,300</td><td>6,0</td><td>0,60</td><td>0,72</td></tr>
<tr><td>4</td><td>42,000</td><td>40,000</td><td>0,300</td><td>29,0</td><td>12,00</td><td>14,48</td></tr>
<tr><td>5</td><td>50,000</td><td>8,000</td><td>0,300</td><td>48,0</td><td>2,40</td><td>2,90</td></tr>
<tr><td>6</td><td>53,000</td><td>3,000</td><td>0,300</td><td>100,0</td><td>0,90</td><td>1,09</td></tr>
<tr><td>7</td><td>53,000</td><td colspan="5">Column Wash</td></tr>
<tr><td>8</td><td>60,000</td><td>7,000</td><td>0,300</td><td>100,0</td><td>2,10</td><td>2,53</td></tr>
<tr><td>9</td><td>60,000</td><td colspan="5">Stop Run</td></tr>
<tr><td>10</td><td>60,000</td><td colspan="5">Column Equilibration</td></tr>
</table>
</td></tr>
</table>

Table S5 MS settings

General	MS1	MS2	General comments; special settings
Lumos<br>Tune v4.1.4244<br>Xcalibur v4.7.69.37<br>SII: 1.7.0.468<br>Gradient: 60 min	Analyzer: FT<br>Res.: 120000<br>SR: 375 - 1500<br>AGC: Standard<br>AGC abs.: 400000<br>AcT: 50 ms<br>RF: 30<br>IsM: Q<br>SF: --<br>DDM: CT/3sec	Analyzer: IT<br>Res./ScR: -/rapid<br>SR: Auto<br>AGC: 300%<br>AGC abs.: 30000<br>AcT: 35 ms<br>CS: +2 to +7<br>IsM: Q<br>IsW: 1.6<br>Frag.: HCD<br>NCE: 32	classic orbitrap experiment: MS1 in Orbitrap at high resolution and data dependent MS2 also in Orbitrap high resolution. Dynamic exclusion enabled (exclude after n times=1; Exclusion duration (s)= 30; mass tolerance= ± 10ppm)<br>Intensity Threshold: 5000<br>Ion transfer Tube Temp: 250 °C<br>Ion Source Voltage: 2500 V

Note: FT= Fourier Transform (Orbitrap); IT= Iontrap; Q= Quadrupol; Res.= max. Resolution at 200 m/z (Lumos) or 400 m/z (Elite) [FWHM (full width at half maximum)]; ScR= scan rate for measurements in the IT; SR= scan range [m/z]; AGC= automatic gain control, max number of acquired ions per measurement; AcT= max. Ion acquisition time [ms]; CS= charge states used for fragmentation; IsM= Isolation mode (Q or IT), MS2 isolation and further is only done in IT; IsW= Isolation window [m/z], value followed by scan mode the isolation is based on (MS1, MS2 …) Frag.= Fragmentation method; HCD= Higher-energy collisional dissociation; CID= Collision-induced dissociation; ETD= Electron-transfer dissociation; EThcD= Electron-Transfer/Higher-Energy Collision Dissociation; sHCD= stepped HCD; NCE= normalized collision energy; cycles: number of MSn recorded or max cycle time; RF= RF Lens [%]; SF= Source Fragmentation [V]; DDM: Data dependent Mode (cycle time in seconds, CT/[s] or number of scans, NS); NS= Number of data dependent scans.

Table S6 MSFragger search

<table>
<tr><td>Program & version</td><td>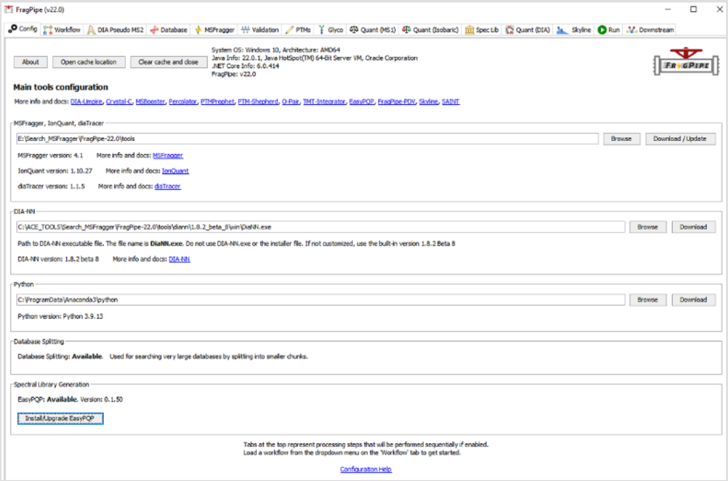
</td></tr>
<tr><td>Search engine</td><td>MSFragger 4.1.</td></tr>
<tr><td>settings</td><td>LFQ-MBR (MBR deactivated); Basically default; MSBooster and DDA (not DDA+)</td></tr>
<tr><td>Static modification</td><td>Carbamidomethyl (C)</td></tr>
<tr><td>Digestion mode</td><td>Trypsin/P (specific), 2 missed cleavages</td></tr>
<tr><td></td><td></td></tr>
<tr><td>Dynamic modification</td><td>Acetyl (N-term); Oxidation (M)</td></tr>
<tr><td>Modification included in quantification</td><td>Oxidation (M)</td></tr>
<tr><td>Databases</td><td>1. 2025-11-14-decoys-contam-ACE_1035_NbLab360.v103.gff3.CDS.fasta.AA_plus_SOI.fasta.fas</td></tr>
<tr><td>Annotation</td><td></td></tr>
</table>

Note: added the sequence of P69B and EPI1 to NbLab360 database and loaded into Fragpipe. Here decoys and contaminants were added.

Table S7 MaxQuant search

Program & version	MaxQuant v 2.7.0.0
Search engine	Andromeda
settings	Basically default; LFQ and no MBR were turned on
Static modification	Carbamidomethyl (C)
Digestion mode	Trypsin/P (specific), 2 missed cleavages
Dynamic modification	Acetyl (N-term); Oxidation (M)
Modification included in quantification	Oxidation (M)
Databases	1. Contaminants<br>2. ACE_1035_NbLab360.v103.gff3.CDS.fasta.AA_plus_SOI.fasta
Annotation	
